# Rating the environmental and genetic risk factors for colorectal cancer

**Published:** 2012

**Authors:** M Toma, L Beluşică, M Stavarachi, P Apostol, S Spandole, I Radu, D Cimponeriu

**Affiliations:** * Department of Genetics, University of Bucharest, Bucharest, Romania; ** “Carol Davila” University of Medicine and Pharmacy, Bucharest, Romania

**Keywords:** colorectal cancer, relative risk, environmental and genetic factors

## Abstract

Colorectal cancer (CRC) is a disease preventable in up to 50% of the patients by lifestyle modifications. The preventive strategy for the decrease in the incidence and mortality of CRC is based on understanding the relations between the environmental and genetic factors. The most important identified risk factors for CRC are aging, personal and familial history of CRC or adenomas, hereditary colon cancer syndromes, dietary patterns, and inflammatory bowel disease. The purpose of this review is to update data referring to environmental and genetic documented factors and CRC risk. Using data from the Medline database, we analyzed reports on CRC risk published between 2000 and 2010. We realized a classification taking into consideration the relative risk (RR) reported for each analyzed factor (RR ranged between 1 and 6.87). The highest RR were represented by the patients with distal advanced cancer (RR = 6.7) and those with high dysplasia adenomas (RR = 6.87). In the future, evaluation and optimisation of screening options will stay at the base of new prevention strategies that will be implemented based on the influence of risk factors identified in each population.

## Introduction

During recent decades, malignancy has become a serious health problem in the European countries, being the second most common cause of death after cardiovascular diseases [**1**]. In 2008, colon and rectum cancers represented 9.8% of the world’s total new cancer cases (about 1 million). Worldwide, in terms of incidence (per 100.000 inhabitants), CRC was ranked as the third malignancy in men (10%) and the second in women (9.4%). Survival estimates at five years after diagnosis for men was 56% in Western and 35% in Eastern Europe, whereas for women was 53% and 36%, respectively [**2**].

CRC represents a disease that can be preventable in up to 50% of cases by lifestyle modifications (i.e. balanced diet, avoidance of smoking and alcohol, moderate physical activity) [**3**]. Understanding the relations between environmental and genetic factors may improve the strategies that are implicated in the decrease of incidence and mortality of CRC.

The purpose of this review was to update the data referring to environmental and genetic documented factors and CRC risk. We analyzed data from reports on CRC risk using the studies of the Medline database published between 2000 and 2010, and we realized a classification taking into consideration the relative risk (RR) reported for each analyzed factor.

### Documented risk factors for colorectal cancer

We selected the factors with a RR above 1.0, which suggested that exposed people have a higher risk of disease than non-exposed persons [**4**]. The most important identified risk factors are aging, personal and familial history of CRC or adenomas, dietary patterns, inflammatory bowel disease, and hereditary colon cancer syndromes [**3**]. Documented risk factors for CRC arranged in order of RR are shown in **Table 1**.

**Table 1.** Classification of risk factors associated with CRC in terms of relative risk

**Risk factors**	**Category at risk**	**RR**	**Ref.**
**Age**	Patients who have proximal advanced cancer	**1.3**	[**6**]
**Socio-economic status**	Patients with low socioeconomic status	2.88/2.42*	[**9**]
**Personal history of polyps**	Patients with distal hyperplastic polyps	**2.6**	[**6**]
	Patients with distal tubular polyps	**4.0**	
	Patients with distal advanced cancer	**6.7**	
**Personal history of CRC**	Patients with 1-2 tubular adenomas <1cm	**1.92**	[**13**]
	Patients with 3 or more tubular adenomas <1 cm	**5.01**	
	Patients with tubular adenomas > or = 1cm	**6.4**	
	Patients with villous adenomas	**6.05**	
	Patients with high dysplasia adenomas	**6.87**	
**Family history of CRC and colorectal adenomas**	Patients with first-degree relatives of rectal cancer	**1.89**	[**20**]
	Patients with first-degree relatives with CRC	**2.25**	
	Patients with a parent with CRC	**2.26**	
	Patients with first-degree relatives with colonic cancer	**2.42**	
	Patients with brother / sister with a common parent having CRC	**2.57**	
	Patients with relatives diagnosed with CRC before the age of 45 years	**3.87**	
	Patients with more than one relative with CRC	**4.25**	
**Personal history of inflammatory bowel disease**	Patients with CRC and UC	**1 – 2.75**	[**25**]
**Diet with high content of red meat and fats**	Patients having a diet rich in red meat	**1.17 / 1.83†**	[**31**]
**Alcohol**	Consummator	**1.56**	[**38**]
**Smoking**	Smokers	**1.20 / 1.38**	[**40**]
**Menopause status**	Pre menopause women	**1.61**	[**47**]
**Sedentary behaviour**	Sedentary patients	**1.61**	[**41**]
**Obesity**	Patients with BMI> or = 40	**2.39 / 1.49†**	[**44**]
**Low-penetrance polymorphisms**	APC I1307K	**1.5 – 2.2**	[**64**]
	TGFβR1*6Ala	**1.2**	[**65**]
	HRAS1	**2.5**	[**58**]

Obs: * Estimated risk for colonic and rectal cancer; **†** Estimated risk for men and women

### 1. Age

CRC incidence increases exponentially after the age of 50 years. The statistics for the period 2001-2005 provided by SEER Cancer Statistics Review shows that the incidence of CRC was 18.4 for persons under 65 years, and 273 for persons over 65 years [**5**]. Age over 65 years represents an independent risk factor for advanced proximal colon cancer. A RR of 1.3 has been estimated for each interval of five years for the age range between 50 and 80 [**6**].

### 2. The ethnic and racial background

The largest value of CRC incidence among different ethnic groups was estimated for Afro-American people (61.2) [**5**]. Ashkenazi Jews present a higher risk for CRC attributed to genetic susceptibility and/or lifestyle [**7**]. For Israeli Jewish males, according to the birth place, CRC incidence was 48.3 for European-American, 35.5 for Asian and African and 32.7 for Israeli [relative risk (RR) 1.36], while the incidence value in women were 35 for European-American and 26 for all others (RR 1.35). Response to adjuvant oncological therapy and survival time after diagnosis may present ethnic differences [**8**].

### 3. Socio-economic status

Recently, a relationship between socio-economic status and incidence of some types of cancer has been estimated; however, connecting paths between them are not fully elucidated yet. It has been showed that low education status is associated with a RR of 2.88 for colon cancer and 2.42 for rectal cancer [**9**]. A study realised in Denmark, showed that the incidence of CRC is associated with greater social disadvantage, predominantly among men [**10**].

### 4. Personal pathological history of polyps and CRC

#### 4.1. Personal history of colorectal adenomas

Regarding the relationship “adenoma – carcinoma”, personal history of colorectal adenomas increases the risk of CRC [**11**]. The main factors for advanced proximal colonic cancer were the characteristics (i.e. size, number and histology) of distal adenomas. For instance, the RR for proximal advanced cancer is 2.6 for hyperplastic polyps, 4.0 for tubular polyps and 6.7 for distal advanced cancer when compared with patients without distal polyps [**6**]. The incidence of CRC could be reduced by polypectomy [**12**].

#### 4.2. Personal history of CRC

Patients with CRC who have undergone curative resection may present new colorectal or other types of tumours. The highest risk of developing new cancers was calculated for patients diagnosed with CRC at an age less than 60 years. The RR calculated for patients with baseline neoplasia is influenced by the size and number of adenomas (i.e. 1.92 for 1-2 adenomas < 1cm, 5.01 for 3 or more tubular adenomas < 1 cm, 6.4 for tubular adenomas ≥ 1cm) and histological type (i.e. 6.05 for villous adenomas and 6.87 for high dysplasia) during the five years period of surveillance colonoscopy [**13**].

Endoscopic evaluation allows detection of synchronous tumours for 4-7% of patients [**14,15**] and of synchronous adenomas for 55% of CRC patients [**14**]. These synchronous cancers were associated with a higher rate of mortality and an increased rate of tumour progression compared with single cancers [**14**].

Patients with CRC also have a high risk of developing metacrone cancers (4%) and adenomas (25%) [**16**]. Recent studies showed that the cumulative occurrence of metacrone CRC was 1.8% at five years, 3.4% at ten years, 6.3% at 15 years and 7.2% at 20 years [**17**,**18**]. Younger age was associated with an increased RR for metacrone CRC, although cumulative rates after 15 years were low [**17**].

### 5. Family history of CRC and colorectal adenomas

The risk of CRC is increased by heredo-collateral history of CRC or adenomatous polyps (i.e. any first-degree relative less than 60 years; history of CRC or adenomatous polyps in two or more first-degree relatives at any age). The risk of a 50 years old person to develop CRC increases from 1.8% to 3.4% if he/she has at least one affected relative and to 6.9% if he/she has more than two affected relatives [**19**].

A meta-analysis confirmed these data and showed that the CRC risk increases if patients were diagnosed at a younger age and increases supplementary if they have more than two affected relatives (the estimated RR compared with those who do not present a family history ranges from 1.89 to 4.25) or present a family history of adenomas (the estimated RR for subjects with first-degree relatives with colorectal adenomas was 1.99) (**Table 1**) [**20**].

### 6. Colorectal inherited syndromes

Five to ten percents of patients with CRC present an inherited disease, the common colorectal syndromes being familial adenomatous polyposis (FAP) and hereditary non-polyposis colorectal cancer (HNPCC) [**21-23**]. Less common hereditary colorectal cancer syndromes are listed in **Table 2**.

**Table 2.** Hereditary Syndromes Predisposing to Colorectal Cancer

**Syndrome**	**Gene**	***Chromosome***	***Type of gene***	**Hereditary Pattern**
***FAP***	*APC*	5q21-q22	Tumor suppressor genes	Dominant
***Lynch***	*MLH1* *MSH2* *MSH6* *PMS2* *EPCAM* *(TACSTD1*)	3p21.3 2p21 2p16 7p22.2 2p21	Repair/stability genes	Dominant
***Attenuated polyposis***	*AXIN2*	17q23-q24	Tumor suppressor genes	Dominant
	*MYH (MUTYH)*	1p34.1	Repair/stability genes	Recessive
***Juvenile polyposis***	*BMPR1A*	10q22.3	Tumor suppressor genes	Dominant
	*SMAD4 (DPC4)*	18q21.1	Tumor suppressor genes	Dominant
***Bloom***	*BLM*	15q26.1	Repair/stability genes	Recessive
***Cowden***	*PTEN*	10q23.3	Tumor suppressor genes	Dominant
***Li-Fraumeni***	*TP53 (p53)*	17p13.1	Tumor suppressor genes	Dominant
***Peutz-Jeghers***	*STK11*	19p13.3	Tumor suppressor genes	Dominant

FAP represents approximately 1% of all CRC and is due to inherited mutations of the APC gene. It is characterized by the presence of hundreds to thousands of polyps throughout the intestine, which are usually diagnosed around the age of 15 years old. Nearly all patients up to 40 years old will develop CRC if they do not practice preventive resection of the colon [**22**,**23**].

HNPCC (known also as Lynch syndrome) represents approximately 3-4% of all CRC and the risk of developing CRC throughout lifetime ranges between 70-80%. HNPCC is caused by the changes in a set of genes which are involved in repairing DNA lesions and it is characterized by early onset of a large number of synchronous and metacrone colorectal tumours, accompanied by extracolonic tumours (i.e. endometrium, kidney, pelvis, urethra) and skin lesions (i.e. adenomas, keratoacantomas, carcinomas) [**22**,**23**].

### 7. Personal history of inflammatory bowel disease

Inflammatory bowel disease (IBD) which includes ulcerative colitis (UC) and Crohn's disease (CD) causes colonic inflammation and predisposes to CRC [**24**]. UC and CD show similar colonic dysplasia around the neoplastic lesions [**25**]. The RR for CRC in patients with UC was estimated to range between 1 and 2.75 [**26**]. The most important risk factors for CRC in patients with IBD are represented by the increased duration of disease, younger age at diagnosis, the extension of inflammatory lesions, primary cholangitis and family history of CRC [**27**,**28**].

### 8. Factors related to lifestyle, diet and environment

The risk for CRC is influenced by various factors related to diet (i.e. low intake of fruits, vegetables and fibre; increased consumption of red meat and saturated fat), lifestyle (i.e. increased body mass index, the lack of physical activity, smoking) and environment (i.e. professional exposure, radiation, drugs) [**29**,**30**]. For example, the RR for a diet rich in vegetables and fruits is 0.81, while for a diet rich in red meat RR is 1.17 in men and 1.83 in women [**31**].

Various studies have demonstrated that an increased serum level of vitamin D and calcium intake decreased risks for CRC [**32,33**]. The high protective effect of increased calcium intake was calculated for tumour location in the distal colon compared with the proximal colon (RR 0.65) [**34**].

Multivitamin use for at least 10-15 years may reduce the risk of developing colon cancer. The lowest risk was estimated for subjects who took >400 µg folic acid/day and for those with history of CRC (RR 0.48) [**35**].

The association between alcohol consumption and CRC is influenced by the quantity of alcohol consumed and not the type of drink [**36,37**]. Compared to individuals who do not consume or consume alcohol occasionally, the RR reported for individuals who consume alcohol was 1.56 [**38**].

Some of the carcinogens present in cigarette smoke can be absorbed and can promote various digestive tract cancers, including CRC [**39**]. It has been reported an increased risk for CRC associated with smoking duration (40 years of smoking duration increase RR to 1.20), daily cigarette consumption (40 cigarettes/day increase RR to 1.38) and pack-years (60 pack-years increase RR to 1.51) [**40**].

Data from epidemiological studies have shown that sedentary behaviour has been associated with CRC (RR 1.61) [**41**], whereas physical activity is protective (RR 0.76-0.79 for men and 0.79-0.85 for women) [**41**,**42**].

Body mass index (BMI) was associated with increased risk for CRC [**43**]. Thus, an increase of five units of BMI elevates the risk for colonic cancer in both sexes (RR 1.12 for women and 1.30 for men) [**44**], whereas a BMI ≥ 40 kg/m^2^ increases the RR to 1.49 for women and 2.39 for men [**45**]. For women, the risk for CRC is influenced by both BMI and menopausal status. In a multicenter randomized controlled trial of mammography screening for breast cancer, which included over 89,000 women aged between 40 and 59 years, a BMI >30 kg/m^2^ increased two-fold the risk for CRC in pre-menopause women compared with those at the menopause [**46**]. It has been shown that for an increase by ten units of BMI, the RR for CRC in pre-menopause women is 1.61 compared with those to post menopause [**47**].

Occupational exposure to non-metallic minerals, leather and textile industries, communications, petroleum products can increase the risk of colonic cancer [**48**,**49**].

Recent epidemiological studies have shown that there is a decreased incidence of adenomas, carcinomas and mortality associated with cancer. Non-steroidal anti-inflammatory drugs (NSAIDs) have been associated with decreased CRC risk (RR between 0.6 – 0.7) [**50,51**].

### 9. Infectious agents and colorectal cancer

It was reported that infectious agents accounted for approximately 18% of all cancers, worldwide [**2**]. Some cancers have known infectious etiologies (i.e. cervical, liver or gastric cancers). Recently, the infectious agents have been taken into consideration as a possible cause of CRC. The problem of the association of infections with common viruses (like Torque teno virus) and the development of colorectal cancer must be tested, although some infectious agents (i.e. *Helicobacter pylori*, *Streptococcus bovis*, *JC virus*, *human papillomavirus*) [**52**] have been correlated with this disease.

Also, there are particularities regarding the evolution of CRC in patients with viral infection. Thus, it was shown that chronic *hepatitis B virus* (*HBV*) infection with viral replication reduces hepatic metastasis of *colorectal* cancer, and therefore prolongs the survival of patients [**53**].

Non-AIDS-defining malignancies have come to represent a growing fraction of the overall cancer burden in HIV-infected people. Anal cancer incidence is especially high due to sexual transmission of HPV and is caused by persistent infection with oncogenic subtypes of HPV [**54**]. Recent data show that *HIV*-infected patients with adenocarcinoma of the colon tend to be young men with a high incidence of right-sided involvement [**55**].

### 10. Genetic predisposition – genetic polymorphisms

Genetic syndromes associated with CRC (FAP and HNPCC) causes 5-10% of all CRC and increase two-fold the disease risk relative to the level of patients with sporadic CRC [**21**]. Regarding colorectal lesions, phenotypically FAP and HNPCC have a great variability, both intra and interfamily [**56**,**57**].

Variation in risk and phenotypic predispositions for CRC disease may be partially explained by the involvement of low-penetrance genes like those involved in metabolization or control of cell homeostasis, genomics stability and progression of cell cycle [**58**-**60**].

Genome-wide association studies (GWAS) allow linkage analysis of hundreds of thousands of SNPs simultaneously, providing a powerful approach for identification of low penetrance alleles that can modify the risk for CRC [**61**].

Since 2007, when the first common low-penetrance susceptibility variant was associated with the risk of CRC [**62**], ten common genetic variants or single-nucleotide polymorphisms (mapped to chromosomes 8q23, 8q24, 10p14, 11q23, 14q22, 15q13, 16q22, 18q21, 19q13 and 20p1) have been linked to CRC [**63**].

A recent GWAS shows that *G* allele of rs6983267 mapped to 8q24 is significantly associated with cancer pathogenesis. Homozygosis for the G allele of this SNP increases CRC risk 1.5-fold (a relatively weak effect), but this allele shows relative copy number increase during tumor development. This region may act as a transcriptional enhancer because it contains a sequence that enhances the MYC expression by binding T cell factor 4 (TCF4) and thus influence Wnt signaling [**63**].

However, the low-penetrance variants have relatively minor effects on cancer risk by themselves. However, the combinations of multiple variants in association with environmental exposures may improve the predictive model for CRC risk stratification [**63**].

Also, variants of high-penetrance loci (i.e. APC, MLH1, MSH2) may play an important role in familial or sporadic CRC (RR for CRC range between 1.2 to 2.5) [**58**-**65**]. Missense variants that determine synthesis of truncated protein of tumour suppression genes may contribute to the pathogenesis of cancer in some ethnic groups. This was supported by the observation that I1307K variant in the APC gene increase twice the risk for the people of European population with Hebrew origin. This variant was associated with a RR between 1.5 and 2.2 for CRC, based on population analysis [**58**,**66**].

Gene polymorphisms implicated in the metabolism of the drugs commonly used in chemotherapy may be involved in clinical response to chemotherapy and related toxicity. Thus, on the one hand, some polymorphisms implicated in metabolism of oxaplatin or 5FU may be associated with a worse response to therapy (i.e. XRCC1 Arg399Gln) [**67**] and, on the other hand, with longer progression and free survival (i.e. MGMT 2535G>T) [**68**]. Also, other polymorphisms may predict the therapy response (i.e. UGT1A1*28 in relation with irinotecan; thymidylate synthase *3/*3 genotype in relation with 5FU) [**69**].

In the physiopathology of CRC are also involved other mechanisms. The importance of the epigenetic silencing of genes has been recognized in CRC, and this has been linked, for example, to a germline hypermethylation of the DNA mismatch repair genes MLH1 and MSH2 that may serve as predisposing events in some CRC patients [**63**].

In addition, implication of several microRNAs that can interact with genes such as K-RAS, APC, p53, PTEN, TCF4, COX-2, DNMT3a and DNMT3b bring new data concerning the role of the genome regions assumed to be ‘junked’ in cancer [**63**].

The new insights about the mechanistic component of initiation and progression in CRC brought by the recent studies will allow understanding the relationship between the environmental and genetic factors in CRC physiopathology.

## Conclusions

Using articles published between 2000 and 2010 from public databases, we realized an update on the risk factors associated with CRC. For the risk factors discussed, RR ranged between 1 and 6.87. The highest RR were found in patients with distal advanced cancer (RR=6.7) and those with high dysplasia adenomas (RR=6.87). In the future, evaluation and optimisation of screening options will stay at the base of new prevention strategies that will be implemented based on the influence of risk factors identified in each population.

## Acknowledgements

This paper is supported by the Sectoral Operational Programme Human Resources Development, financed from the European Social Fund and by the Romanian Government under the contract number POSDRU/89/1.5/S/64109.
